# The Operative Treatment of Pleural Effusions

**Published:** 1874-07-15

**Authors:** William Pepper


### THE OPERATIVE TREATMENT OF PLEURAL EFFUSIONS.

READ BEFORE THE PHILADELPHIA COUNTY MEDICAL SOCIETY, MARCH 25, 1874. BY WILLIAM PEPPER, M. D.

*From the Philadelphia Medical Times.*

IN announcing that I should this evening present a few remarks upon the subject of pleural effusions, I have felt that it would be desirable to pass over the somewhat theoretical questions which may be raised about the diagnosis of these effusions, and to limit my remarks to the more practical and interesting point of their treatment. * * * But few distinct clinical histories remain to us from the earlier days of paracentesis; but doubtless, to judge from the difficulty which even now attends the diagnosis in some cases of pleural effusion, our bold forefathers must have made many a "dry tap."

The more recent history of the operation is somewhat curious. Despite the many instances in which it was known to have been successfully employed, despite the improvements in the mode of operating, and the vast improvement in our power of determining the exact location and extent of the effusion, paracentesis continued to be regarded as a daring operation, to be performed only under conditions of urgent danger. It is true, when Læennec announced his immortal discovery, he did not fail to perceive the bearing which physical diagnosis had upon the operation under discussion, and he asserts "that paracentesis will become much more common and much more useful in proportion to the diffusion of the employment of mediate auscultation." That this prediction was not more speedily verified is to be explained by the fact that there was still wanting a clear analysis of the clinical symptoms which furnish the chief indications for the operation, as well as the sole guide in the prognosis of its results.

The great credit which is due to Trousseau in connection with paracentesis is, then, undoubtedly this, that in 1843 he for the first time gave a clear and impressive account of the precise indications which call for the operation, while at the same time he simplified the operative procedure, and, by his great success in repeated instances, confirmed his precepts by the most extended practice. Becker, Schuh and Skoda had, a few years previously, published valuable memoirs, which anticipated most of Trousseau's conclusions; but it was reserved for this latter teacher, by his firmness and eloquent advocacy and brilliantly successful employment of paracentesis, to bring the operation into the high favor which it has since enjoyed. On the continent of Europe, its discussion has been ever since "the order of the day;" and in England it has been frequently advocated and performed.

In America, despite the valuable memoirs of Bowditch, published over twenty years ago, the operation has apparently never been so popular and generally practiced as abroad. It may, therefore, not be deemed inappropriate if I give a brief sketch of the operation as it is performed by different operators, and as I have myself frequently employed it. * * *

The first method to which I shall allude may be called that of Trousseau, although the chief peculiarity, the valve in the canula, was the introduction of Reybard (*Gaz. Med.*, January, 1841). As described in his work on Clinical Medicine (Syd. Soc. Trans., vol. iii, p. 270), the apparatus consists merely of a bistoury, with which an incision is made through the skin at the point to be punctured, and a common trocar, the lips of which are surrounded with a gold-beater's skin which is softened by being wetted. When gold-beater's skin cannot be obtained, a piece of the intestine of a fowl, rabbit, or cat, or a bit of bladder, will serve the purpose of forming a valve which will allow the fluid to flow out during expiration, whilst during inspiration it rests in exact apposition to the expansion of the canula and prevents the entrace of air to the chest.

A modification of this method, which I have employed in many cases with entire success, is as follows: A piece of thin India-rubber tubing is attached to the canula, and the trocar is passed through the tubing before entering the canula. The free end of the tubing is placed in a vessel containing a little water. After the puncture has been made the stylet is withdrawn, the minute hole in the elastic tubing instantly closes, and the fluid escapes into the vessel, the water of which effectually prevents any return of air during the interruption of the flow of the fluid. * * * I have already alluded to the fact that so long ago as the time of Scultetus the use of a syringe was recommended to exhaust the portion of the effusion which was below the level of the external opening. Lænnec advised the use of a cupping-glass and exhausting-syringe, with the view of draining off the last remains of fluid and of facilitating the expansion of the lung. But more recently, greatly improved suction-pumps or aspirators have been devised. Among these, the apparatus devised by Bowditch deserves prominent mention. And still more recently, Dieulafoy has modified the syringe by introducing a stopcock between the nozzle and the chamber, so that the latter may be exhausted, and then, after the point of the trocar is buried beneath the skin, the vacuum may be brought into connection with the cavity of the needle and the point be endowed with a spontaneous power of suction.

He has also devised a modification by which a vacuum is created in a graduated jar, and the cavity of the needle brought into connection with this, so that the amount of fluid withdrawn can be directly observed. This latter form has the disadvantage that in case of large effusions it is necessary from time to time to detach the receiver (which contains about twenty fluidounces), empty it, renew the vacuum and re-attach it. Where the collection of fluid to be withdrawn is small, and particularly if it be also offensive, this instrument enables you to remove it without exposing it to the atmosphere at all.

In attempting to estimate the relative advantages of these various methods, I would not be understood as disparaging the merits of any, whilst at the same time I would raise my voice against the unquestioning, unmeasured laudation which has been bestowed in many quarters upon Dieulafoy's apparatus. The sole object which is to be accomplished by paracentesis is the withdrawal of the effusion without permitting the entrance of air. In considering which of the above methods best secures this purpose, it is to be first observed that they all provide effectually against the possibility of the entrance of air during the withdrawal of the fluid. Let us ask, in the next place, by what forces the removal of the fluid is to be accomplished. In employing either of the two first modes, where a simple puncture with a guarded canula is made, there is no external power whatever employed. The forces which expel the fluid are —1, gravity, which leads so much of the effusion as is above the level of the external opening to escape, in seeking its level; 2, the tendency of the chest-wall, which has been greatly stretched, and of the adjoining viscera, which has been displaced, to return to their normal limits, by expelling a portion of the effusion; and 3, the centrifugal pressure of the expanding lung.

It is further evident that the first force, that of gravity, will be able to operate directly in proportion to the activity of the two latter elements, since, if the lung be entirely non-expansive, the chest-wall rigid and inelastic, and the dislocated viscera fixed in their morbid position, there will be little or no escape of fluid. In other words, the rapidity and the extent of the withdrawal of the effusion are measured by the promptitude and degree of the return of the viscera to their normal condition. It will therefore always be observed that the fluid, which at first flows in a steady stream, soon begins to escape by jets corresponding to the movements of respiration.

On the other hand, in the employment of either of the two later methods—Bowditch's syringe, or Dieulafoy's aspirator—we bring to bear an additional power—, that of the suction of a vacuum. In regard to the aspirator, it must be remembered that it possesses one virtue peculiar to itself, and which gives it a high diagnostic value. As there is constantly the full suction-power of the vacuum at the end of the needle as it is pushed through the tissues, it follows that the instant the fluid is reached it will be seen to enter the chamber of the syringe. Whereas, it is quite possible that, in case of a comparatively thin layer of fluid included between pleuræ thickened by plastic deposits, the trocar of a Bowditch's syringe or an exploring-needle might be passed through the liquid stratum and have its point imbedded in the thickened pulmonary pleura, and thus seriously mislead the operator. But apart from this special diagnostic value, and of its great importance in some cases I am well aware, Dieulafoy's aspirator has no advantage over Bowditch's syringe, while it has the disadvantage that, as the barrel of the syringe is small, and the piston necessarily works very tightly, the evacuation of a large collection of fluid is both tedious and fatiguing. Both instruments are, however, alike in this, that when they are used, instead of the fluid being expelled by the forces we have already considered, it is sucked out by a force which varies with the perfection of the vacuum created in the syringe, but which is in all cases quite considerable. When, therfore, all the conditions exist which render it quite impossible for the parts to return quickly to their normal position—when the lung is tightly bound down, and the chest-wall rigid—it is quite possible, despite Dieulafoy's assertion to the contrary, that an injurious traction may be exerted upon the lung by the forced withdrawal of fluid from the pleural sac. * * *

It must be further asked, in connection with this point, how much advantage attaches to the entire withdrawal of the effusion? It has been held by some that it is undesirable to do so, but I confess to being unable to perceive any good reason for fearing to do it in cases where it is evident, by the return of pulmonary resonance and the development of the vesicular murmur, that the lung is expanding freely to occupy the place of the effusion. In such cases it is undoubtedly possible to withdraw the accumulation by the simple canula, as described. In other instances, where the effusion is serous, but yet the lung is incapable of fully expanding by the mere pressure of the atmospheric air entering its tissue, it may be of service to supplement this by the force of aspiration. But I have not observed in such cases any disadvantage from allowing a portion of the effusion to remain, since it has often been possible, after the excessive distention was removed, to secure the absorption of the remainder as the lung gradually expanded.

In fine, the result of my own experience has been that the greatest value of the "aspirator, with the previous vacuum," in cases of pleural effusion, is a diagnostic one; that in cases of large effusions, when the lung is free to expand, the effusion can be easily, safely, and successfully withdrawn by a simple guarded canula; that in cases where the inability of the lung to expand prevents free escape of the fluid through the canula, it is desirable to employ an exhausting syringe, unless its use is attended by such severe pain as to indicate excessive tension upon the pulmonary tissues or upon organized adhesions.

---

BROOKLYN CITY HOSPITAL REPORTS.—*Inhalation of Chloroform in a case of Strychnine Poisoning.*—Patient took five (5) grains of strychnine with suicidal intent.

Before admission to the hospital he was given twenty grains of sulphate of zinc with effect. He had repeated convulsions, and, while being taken from the ambulance, was seized with one of tetanic form, which plainly showed strychnine poisoning. Every muscle was rigid, and tetanus complete. Opisthotonos, irregularity of pulse, varying from 120 to 140 in the minute, with all the accompanying symptoms, were noticeable.

He was immediately placed under the influence of chloroform. The convulsions ceased from the commencement of the anæsthesia, under which the patient was fully kept for three (3) hours. The chloroform was then removed, but the patient did not awake until six (6) hours afterwards—a case of recovery.

*Chronic Diarrhœa.*—In those cases where the epithelium is stripped from the tongue, and the patient presents the cachexia of the disease, good results have been obtained by the administration of pulv. ipecac, in twelve-grain doses, three (3) times daily, given mid-time between meals to prevent emesis.

This is continued until the stools are of a perfectly serous nature, when the ipecac is discontinued, and

Zinci oxid. . . . . . . . . . gr. iv.
Ext. quassiæ . . . . . . . . gr. vj.

in capsule given three times daily.

Guarana in powder has been used in similar cases with apparently very good cures; but as it is impossible to keep trace of the patient, the permanency of the cure is not established.

*Peculiar Injury of Finger.*—A somewhat peculiar case was brought to our notice of a machinist, who, while at work, had the end of his index-finger caught in the machinery.

The ungual phalanx was torn off, and attached to it was thirteen inches of the flexor tendon, and muscular connection. No inflammation of the arm ensued.

*Night Sweats.*—For night sweats of phthisis the combination of extract of belladonna with oxide of zinc:

Belladonnæ ext........gr. $\frac{1}{4}$

Zinci oxid. ...........gr. iv.

is found acceptable.

*Excision of portion of Sternum.*—This is a case where the greater portion of the manubrium was excised, by Dr. Spier, for necrosis.

*Rupture of Lung.*—Patient, seventeen years of age, was suddenly caught and crushed between two cars, resulting in fracture of neck of humerus, and death from emphysema. Autopsy revealed rupture of left lung through apex, four and a half inches in depth. No fractured rib to cause it; but the lungs were filled, and in the sudden pressure the air was not able to escape by the natural passage; a rupture was the consequence.—*N. Y. Med. Record.*

---

THE ABDOMINAL BRANCHES OF THE PNEUMO-GASTRIC NERVES AND THEIR RELATION TO THE TREATMENT OF CHOLERAIC DISCHARGES.—The subject of the Annual Essay read before the Minn. State Med. Society by H. C. Hand, M.D., (*Northwestern Med. and Surg. Journal.*) This is an article to which we refer with especial pleasure, since, while it contains a few excellent suggestions, theoretically deduced, it does not leave the domain of facts to enter that of speculation. Epitomizing from the lengthy original, we deem it unnecessary to quote the author's experiments and dissections, as they go mainly to confirm the researches of others. First describing the anatomical distribution of the vagus, he enters into the physiology of that wonderful nerve, limiting his remarks chiefly to its action on the abdominal viscera. The various experiments on the subject, up to date, are carefully cited, and their general results, as well as those of the author's vivisections, which, few as they were, were much interfered with by the meddling of some anti-cruelty people, show pretty evidently the antagonism between the vagus and the great sympathetic, and the increase of secretory action on excitation of the former, as well as the diminution of transudation and gland activity consequent upon section of the pneumogastrics. Waller's experiments on the human vagus are referred to, by compression of which, resulting when intense, in a temporary paralysis, he had repeatedly controlled obstinate vomiting; but more than this, Horatio C. Wood, Jr., found that section of that nerve in animals, counteracted the effects of the most powerful emetics and cathartics almost invariably, the autopsy proving the gastric and enteric mucous lining dry and mostly pale.

The two morbid processes, vomiting and diarrhœa, being thus dependent on vagal integrity, the question is raised by Dr. Hand, whether their inordinate continuation ought not to be interrupted, as we are aware that we can do this, by temporary paralysis of those nerve-tracts? The greater influence of the left vagus on the stomach, and of the right on intestinal action would indicate sedation of the former in vomiting, of the latter in choleraic discharges. As to the means of sedation, cold applied to the side of the neck would seem a more effective and preferable measure than Waller's compression, which is at the best difficult to perform; but in as intractable a disease as cholera, would not even a harsher method be justifiable to save life? Section of one vagus is, in animals, not a dangerous operation; indeed, division of both nerves is by no means always fatal, and comparative physiology has failed to point out any greater danger from that operation in man, as cases of traumatic injury of that nerve have confirmed; now, as union of the divided ends is of speedy occurrence, when care is taken to prevent any separation of the extremities, surely another chance for life ought not to be refused to the patient, dying of intestinal discharge, and section of the right vagus ought to be given a trial, as a last resource in extreme cases. With these few remarks we will close, referring for further information to the interesting original.

---

PARALYSIS OF THE RADIAL NERVE. Panas (*Arch. Gen. de Medicine*, *Gaz. Med. Ital.*, No. 35, 1873, and *Allg. Central Zeitung*) has, after observation of numerous cases of radial paralysis, been unable to agree with the opinion of other physicians that this paralysis is brought about through cold, and is of rheumatic origin, but holds rather that it is due to pressure on the nerve. From seventeen cases he draws the following conclusions:

1. Paralysis of the radial nerve, in most, if not in all cases, is produced by a light persistent pressure on the nerve.

2. This pressure takes place on a superficially situated portion of the nerve, and the paralysis is limited by this portion.

3. The cause of the pressure is usually the weight of the body, more especially the head, for which the arm forms a support.

4. The longer the pressure has been continued, so much the more apparent will be the paralysis.

5. The paralysis comes on after a long and deep sleep.

6. Rapid and severe fatigue, which causes a lethargic sleep, favors the production of this paralysis.

7. Sometimes in slowly developed and progressive cases, the causes are unknown.

8. In thirty cases of radial paralysis which came under the observation of the author in the clinic and his private practice, in none could he discover rheumatism as a cause.

9. The anatomy of the parts as well as the etiology, the physiology, and the symptomatology, point to a mechanical disturbance of nerve function and consequent paralysis.

10. Rheumatic paralysis, due to cold, does not present the peculiarities afforded by radial paralysis, caused by compression.

11. Electricity cures the most of these paralyses if the disease is not of long standing, and the nerve, upon which the electric contractility of the muscles depends, has suffered no alteration.

---

THE EFFECT OF ALCOHOL ON DIGESTION.—In his lectures "On the Functional Derangements of the Liver," recently delivered at the Royal College of Physicians, Dr. Murchison gave the following sketch of the effect of the habitual use of alcohol on digestion: "Gradually the patient is taught by experience to become more careful as to what he eats and drinks. One thing after another he is obliged to give up. First, he renounces malt liquors; then he discovers that Port wine, Madeira, Champagne, and Burgundy disagree, and he betakes himself for a time to 'dry sherry;' but at length this does not suit, and after an interval, during which a trial is made of claret or hock, the patient, probably under medical advice, finds temporary relief from the substitution for wines, of brandy or whisky largely diluted with water. At last, unless he be misled by the fashionable, but to my mind erroneous, doctrine of the present day, that alcohol in one form or another is necessary for digestion, or to enable a man to get through his mental or bodily work, he finds that he enjoys best health when he abstains altogether from wines and spirits, and drinks plain water."—*Student's Journal and Hospital Gazette.*

---

MENINGITIS TUBERCULOSA.—J. A. Waldenstrom, of Upsala, gives, (*Deutsch Klinik*, 1873, No. 29,) as the two characteristic symptoms to aid in the diagnosis of this disease in early infancy, the vomiting, and the condition of the fontanella. When the former occurs after awakening or during sleep and after change of position, from lying to sitting, or the reverse, especially shortly after eating, abdominal difficulties being at the same time excluded, it can generally be considered as due to irritation of the great nervous centres and affords a suspicion of tuberculous meningitis.

The other principal aid to the diagnosis in very young infants, the protrusion of the fontanella, is due to the intra-cranial pressure from the effusion into the ventricles. This symptom, which is generally overlooked by the text books, has been constant in all the cases observed by the author, though not always equally easy of detection.

In one case where chronic catarrh of the stomach and bowels, with vomiting and diarrhœa co-existed, he diagnosed the disease by this symptom alone, and though doubted by his colleagues of the Poliklinik, the correctness of his judgment was verified by the autopsy.

The diagnosis without these symptoms may be sufficiently easy to the skilled practitioner, but those less accustomed to diseases of children, will find them useful, especially so as they occur at a period when no knowledge of his or her subjective sensations can be obtained from the little patient.

---

CHOLERA.—An international "cholera congress," according to the *Eastern Budget*, is to meet at Vienna next month. Among the most important questions to be discussed on this occasion are the following:—1. Is cholera developed in India spontaneously, and is it always produced in other countries by transmission from abroad? 2. Is cholera capable of being transmitted by travelers from one country to another? 3. Can it be transmitted by articles used by cholera patients? 4. Can it be transmitted by provisions? 5. Can it be transmitted by living animals or the corpses of animals who have died from cholera, merely through the medium of the atmosphere? 6. Has the admission of fresh air to a cholera-producing agent any influence on its contagious or infectious properties? 7. How long is the period of incubation in cases of cholera-infection? 8. Are there any means of disinfection by which the cholera-producing or spreading agent may be made positively harmless, or at least weakened with any prospect of success? 9. Should quarantine establishments, to prevent the spread of cholera, be introduced on rivers, land, or sea? 10. Should permanent or temporary international stations for the study of infectious diseases, and the means of avoiding them, be established?—*London Globe.*

